# Variability of Female Responses to Conspecific vs. Heterospecific Male Mating Calls in Polygynous Deer: An Open Door to Hybridization?

**DOI:** 10.1371/journal.pone.0023296

**Published:** 2011-08-24

**Authors:** Megan T. Wyman, Benjamin D. Charlton, Yann Locatelli, David Reby

**Affiliations:** 1 Mammal Vocal Communication and Cognition Research, School of Psychology, University of Sussex, Falmer, United Kingdom; 2 Department of Cognitive Biology, University of Vienna, Vienna, Austria; 3 Réserve de la Haute Touche, Muséum National d'Histoire Naturelle, Obterre, France; University of Maribor, Slovenia

## Abstract

Males of all polygynous deer species (Cervinae) give conspicuous calls during the reproductive season. The extreme interspecific diversity that characterizes these vocalizations suggests that they play a strong role in species discrimination. However, interbreeding between several species of Cervinae indicates permeable interspecific reproductive barriers. This study examines the contribution of vocal behavior to female species discrimination and mating preferences in two closely related polygynous deer species known to hybridize in the wild after introductions. Specifically, we investigate the reaction of estrous female red deer (*Cervus elaphus*) to playbacks of red deer vs. sika deer (*Cervus nippon*) male mating calls, with the prediction that females will prefer conspecific calls. While on average female red deer preferred male red deer roars, two out of twenty females spent more time in close proximity to the speaker broadcasting male sika deer moans. We suggest that this absence of strict vocal preference for species-specific mating calls may contribute to the permeability of pre-zygotic reproductive barriers observed between these species. Our results also highlight the importance of examining inter-individual variation when studying the role of female preferences in species discrimination and intraspecific mate selection.

## Introduction

According to the biological species concept, reproductive isolating mechanisms are integral to the process of speciation [1]. Pre-zygotic (pre-mating) isolating mechanisms often include species-specific signals in the context of mate attraction (to locate and identify appropriate mates) and mate competition (to identify and defend against potential mating competitors). When related allopatric species are brought together, breakdowns in accurately deciphering these species-specific signals can occur, leading to reduced reproductive barriers and increased hybridization [2]. In the case of mate choice, hybridization or introgression can occur if a female (or male) fails to correctly discriminate between the mating signals of conspecifics, heterospecifics, and hybrids, and ultimately mates with a non-conspecific. Hybrid matings can often reduce the reproductive success of the individuals involved through the loss of other mating opportunities and infertile or less fit hybrid offspring [3], [4], although hybrid offspring with increased fitness have also been documented [5]. Ultimately, hybridization and introgression play an important role in evolutionary processes either through species diversification or stabilization [3], [6], [7] with roughly 10% of animal species and 25% of plant species capable of hybridization [8]. Investigating the behavioral mechanisms involved in hybridization is therefore crucial both for conservation applications and a better understanding of speciation processes [2], [9].

Acoustic signals can play an important role in the process of mate choice, functioning in both intraspecific mating decisions (intersexual selection) and interspecific mating decisions (species discrimination) [10], [11], although these processes need not be mutually exclusive [12], [13]. While the role of intersexual acoustic signals in species or subspecies discrimination has been evidenced in insects [14], [15], anurans [16]–[18], and birds [19]–[21], to our knowledge equivalent roles have never been investigated or identified in mammals, despite the existence of very strong interspecific diversity that characterizes the male sexual calls of several groups, such as deer [22] and seals [23].

This study examines the possible role of acoustic communication in hybridization between two species of Cervinae: European red deer (*Cervus elaphus*) and Asian sika deer (*Cervus nippon*). Although closely related [24], [25], these deer have several strong phenotypic differences in both appearance and behavior [26]. Introductions of sika deer into Europe have resulted in hybridization and introgression [27], [28], documented both in captivity [29] and in the wild [30]–[34]. Genetic evidence from wild populations in the UK suggests that hybridization events are relatively rare, and although they can occur in either direction (male sika deer with female red deer or vice versa), the most common direction is between male sika deer and female red deer [34], perhaps initially as a result of male sika dispersing into new areas with few female sika [30], [34]. After hybridization, extensive introgression can occur as the fertile hybrid offspring can backcross with either parent species [30], [34]. Additionally, observations of sika and red deer interactions in areas where both species are present suggest that some male sika deer may gain closer access to female red deer by ‘sneaking’ into red deer harems without incurring active aggression by harem-holding male red deer [35] (R. Putman, personal comm).

Red and sika deer are both polygynous species; male red deer typically defend female harems [36] while male sika deer typically defend territories where females congregate [26], [37]. Both species have evolved highly dimorphic and conspicuous male acoustic signals which are thought to be sexually selected through the mechanisms of mate choice and male competition [36]–[43]. Recent studies on male red deer roars have specifically highlighted the importance of spectral components in affecting female mate choice behavior [43], [44]. In male sika deer, the ‘moan’ is the most prominent call of the rut and although its function has not been specifically tested, it is believed to serve a similar function to male red deer roars [40]. Male red and sika deer loud calls differ very widely in both their temporal patterns of delivery and their spectral structure, reflecting underlying anatomical differences. Male red deer roars have a relatively low fundamental frequency (F0), short duration, and occur in bouts of one to 11 roars per bout (mean F0 = 106.9 Hz, F0 range = 61.7–136.8 Hz, [41]; mean duration = 1.9±0.5 sec, [45]). In contrast, male sika deer moans occur as single calls and have a relatively high and prominent F0 over most of the call and longer durations (F0 range = 196–1187 Hz, mean duration = 4.36±0.23 sec, [40]). Additionally, resonant frequencies called formants are prominent features of male red deer roars [40] but are less salient in male sika deer moans, especially in the high pitched portions of the call where the harmonic spacing does not provide enough spectral resolution to highlight individual formant frequencies. Anatomically, male red deer have a relatively large, descended larynx which is lowered towards the sternum during vocalizations [46], while male sika deer have a comparatively small larynx which does not appear to be descended or mobile (D. Reby, personal observation). Such differences in male mating vocalizations and their underlying vocal production anatomy suggest that these loud calls should contribute to species discrimination, and thus reproductive isolation, in areas where red and sika deer become sympatric.

Here, we investigate the potential role of male vocal behavior in the red×sika hybridization process, by conducting playback experiments using two-speaker choice tests of conspecific (red deer) vs. heterospecific (sika deer) male mating calls on female red deer during estrus and comparing the females' behavioral reactions to calls from these two species exemplars. Given the strong acoustic differences that characterize the species-specific male mating calls of these two species, we predicted that female red deer should show more attention and preference towards the conspecific red deer roars over the heterospecific sika deer moans.

## Methods

### Location and Subjects

Playback trials were conducted during the 2008 breeding season (August 28–29) at the Institut National de la Recherche Agronomique (INRA) Redon Experimental Farm, Clermont-Ferrand, France. Twenty female red deer of reproductive age (age range = 2–15 years old) were used in the experimental trials. This work follows the Association for the Study of Animal Behavior/Animal Behavior Society guidelines for the ethical use of animals in research, and was carried out under the procedural and ethical authorization of the French Ministry of Agriculture (authorization number A37801 to Redon Experimental Farm).

### Protocol for Synchronizing Estrus Cycles

Female reactions to male mating calls may be influenced by the hormonal state of the female [47]. For example, whereas previous work on red deer showed no preferences for F0 variants in peri-estrous females [39], [48], subsequent experimental tests on estrous females revealed strong preferences for roars with higher F0s [44]. Consequently, we synchronized the estrous cycle of all the females so that playbacks could be conducted when they were in peak estrus. Estrous cycle synchronization was initiated by the insertion of intra-vaginal sponges (2×45 mg, Intervet, Angers, France) filled with fluorogestone acetate (FGA). The sponges provided a steady, continuous release of progesterone that inhibited normal hormone cycling by preventing follicular growth and the subsequent release of estradiol. After 12 days, sponges were removed and females were injected with 400 UI of PMSG (pregnant mare serum gonadotropin) in order to induce estrus and ovulation. Playback experiments were conducted during the predicted window of peak estrus, 35–48 hours after sponge removal and injection of PMSG (see [44] for details).

The 20 females used in this study were randomly separated into two groups of 10 females each (Group A and Group B). Group A underwent the protocol for estrus synchronization (and the subsequent playback experiments) one day before Group B.

### Creation of Playback Stimuli

Playbacks were created using mating calls recorded from four different male red deer and four different male sika deer. All male exemplars used in this study are unfamiliar to the current experimental subjects. Roars from four adult male red deer were recorded at Redon in 1996 using a Telinga pro-III-S/(DAT) microphone used without the parabolic reflector (flat frequency response = 80−16 kHz±3 dB) and a DAT Sony TCD-D7 recorder (amplitude resolution = 16 bits, sampling rate = 48 kHz) at distances of 2–10 m. Moans from four adult male sika deer were recorded in 2007 at a farm in Waterford, Ireland using a Sanken CS-1 directional condenser microphone (flat frequency response = 50−20 kHz±3 dB) and a Fostex FR-2 digital field recorder (amplitude resolution = 16 bits, sampling rate = 44.1 kHz). Male red deer roars were re-sampled to 44.1 kHz and the intensity all red and sika deer calls were normalized to 98% of maximum intensity using Cool Edit Pro 2.0 (Syntrillium).

Calls were arranged into bouts (or groups) of calls for each male exemplar. To create playbacks that mimicked natural calling patterns for each species exemplar, red deer bouts consisted of one to four roars (separated by 0.5 sec) while sika deer bouts contained only one moan.

**Figure 1:**
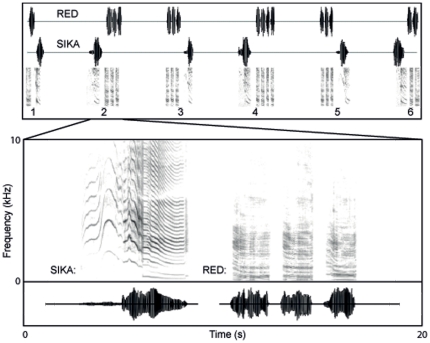
Sample playback sequence. Top: waveforms and spectrograms of the combined playback sequences used to stimulate a vocal exchange between a male red deer and a male sika deer. Bottom: enlarged waveform and spectrogram contrasting the spectral structure of sika deer and red deer vocalizations.

Playbacks consisted of paired sequences of consecutive calling bouts from both species exemplars. Each playback contained six call bouts from both species exemplars, broadcast in a paired dyadic fashion from two different speakers (one speaker played male red deer bouts while the other played male sika deer bouts). The ‘leader’ of each bout pair was alternated for each consecutive pair. The individual bouts within a matched pair of consecutive bouts were separated by 2 sec while bout pairs were separated by 20 sec (Fig. 1). A Latin square design was used to pseudo-randomize 1) which individuals were in each playback sequence, 2) which species was the initial leader of the playback sequence, and 3) which speaker broadcast each species.

The total duration of all calls from each species played during the playbacks was equalized so that females were exposed to each species for a similar total amount of time. Each individual red deer playback contained 16 roars (constructed from a pool of 11 different roars with no roars repeated consecutively or within the same bout and no roars used more than twice) arranged in six calling bouts with an average bout duration of 4.93 sec and an average total call duration of 29.59 sec per playback. Each individual sika deer playback contained six different single moan bouts with an average bout duration of 4.96 sec and an average total call duration of 29.81 sec per playback. Overall, the total duration of each dyadic playback was approximately three minutes.

### Playback Protocol

In a roughly triangular shaped experimental enclosure (approx. 250 m^2^), two Anchor Liberty 6000HIC amplified speakers were hidden behind mesh screens (2 m high×4 m wide) on two ends of the enclosure, facing the entrance (enclosure similar to [41]). The speakers were elevated to 1.5 m above the ground and were connected by coaxial cables to an Apple Macintosh iBook G4 computer used to play the prepared playback sequences. In front of each speaker, proximity zones (each approx. 75 m^2^) were demarcated with white chalk lines starting at 8 m outward from the speakers.

Each playback trial was run on single individuals with each female involved in only one playback trial. At the start of each trial, a female entered at one end of the enclosure near pre-placed food to standardize the starting location and context of the trial. Playbacks were initiated once the subject was equidistant from the two speakers, either standing near or eating the food. Calls were broadcast at levels of 105 dB(C) SPL at 1 m as measured by Radio Shack Sound Level Meter set for C-weighted fast response. Female behavior was video recorded with a Sony Mini DV DCR-TRV19E camcorder from a hidden, elevated observational room.

### Behavioral and Statistical Analysis

We predicted that females would show more attention and preference towards conspecific calls than heterospecific calls. ‘Attention’ was measured as looking towards the speakers and ‘preference’ was measured as entering proximity zones in front of the speakers. The number of instances and the cumulative duration of these two behaviors were quantitatively coded using the digital video analysis software Gamebreaker 7.0.121 (SportsTec, Sydney, Australia) at a 25 fps. Behaviors were coded from the initiation of the playback sequence until 2 min after the last call was broadcast. Looking towards a speaker was defined as occurring when the animal was in a standing position or if moving, when the animal stopped within two steps of initiating the look. A look began when the head first started to orientate to a fixed position facing the speaker and ended when the head started moving away from this fixed position. Entering a proximity zone was defined as starting when the first front leg breaks the outer plane of the zone boundary (white chalk line) and ending when the last front leg passes out of the zone boundary. Video analyses were carried out by MTW with 20% of trials (n = 4) double-coded by DR, resulting in an overall agreement of 99.9% (r_s_ = +0.999, *p*<0.01) on all behavioral measures (and 98.3% agreement, r_s_ = +0.983, *p*<0.01, when excluding cases with all zero values). Because we could not normalize the data distribution for all the response variables, two-tailed Wilcoxon matched-pair signed-rank tests were used to determine if significant differences were present between the behavioral responses of the females to playbacks of the two species exemplars. All statistical tests were performed using SPSS (SPSS for Windows, Rel. 18.0.0. 2009. Chicago: SPSS Inc.) and 0.05 levels of significance are quoted.

## Results

**Figure 2:**
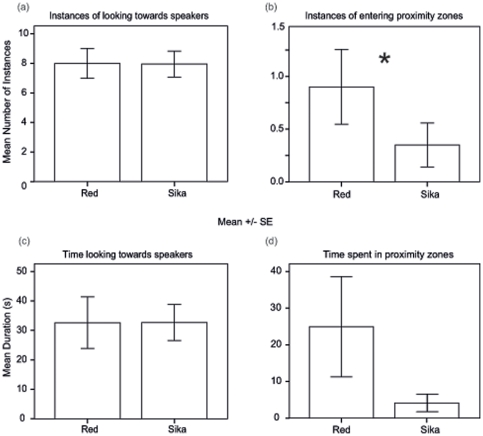
Behavioral responses of focal females to playback stimulus. Error bar charts showing means +/− SE of female red deer behavioral responses to male red and sika deer playback stimulus (*p<0.05; p-values are from Wilcoxon matched-pair signed-rank tests; n = 20); (a) instances of looking towards speakers, (b) instances of entering species proximity zones, (c), time looking towards speakers and (d) time spent in proximity zones.

There was no significant difference in the number of looks given by females towards red deer roars or sika deer moans, (to red deer: 8.00±1.02; to sika deer: 7.95±0.88; z_20_ = −0.09, *p* = 0.93) or in the total duration of looks given by females towards red deer roars or sika deer moans, (to red deer: 32.62±8.75 sec; to sika deer: 32.69±6.15 sec; z_20_ = −0.90, *p* = 0.37) (Fig. 2). However, female red deer entered the proximity zone in front of the speaker broadcasting male red deer roars significantly more times than they entered the proximity zone in front of the speaker broadcasting male sika deer moans (to red deer: 0.90±0.36; to sika deer: 0.35±0.21; z_20_ = −2.43, *p* = 0.015) (Fig. 2). Interestingly, there was only a non-significant trend for females to spend more total time inside of the red deer proximity zone over the sika deer proximity zone (to red deer: 24.92±13.67 sec; to sika deer: 4.13±2.39 sec; z_20_ = −1.69, *p* = 0.091) (Fig. 2), with two females spending more total time in the sika deer proximity zone over the red deer proximity zone (28.25 sec in sika deer zone vs. 25.37 sec in red deer zone and 38.06 sec in sika deer zone vs. 28.56 sec in red deer zone).

## Discussion

Our experiment simulated a situation where allopatric species are brought together and females in estrus are confronted with an unfamiliar species with whom they can hybridize. While these females had been exposed to male red deer and their roars (although not with the specific male red deer used in the playbacks), they had never been exposed to male sika deer. Thus, the playbacks presented during each trial contrasted a familiar conspecific stimulus (male red deer roars) with an unfamiliar heterospecific stimulus (male sika deer moans).

While female red deer showed no significant difference in attention behaviors between the novel heterospecific and the conspecific stimulus, they entered the conspecific proximity zones significantly more times than the heterospecific proximity zones, indicating an overall preference for conspecific calls. There was also a non-significant trend (*p* = 0.091) for females to spend more total time in the conspecific proximity zone. Mating preferences for conspecifics over heterospecifics is well documented [17], [49]–[51], although there can be interspecific or intersexual variation in the directionality of such preferences within hybridizing species [20], [21], [52]. We hypothesized that the very different spectral properties of the sexual calls that characterize these two closely related deer species might contribute to species discrimination, and consequently expected that female red deer would strongly discriminate between the two species by showing a significant preference for conspecific calls. Although our preference hypothesis was supported by predominant female approaches to conspecific speakers, this was not the case for all animals involved in this study as two females spent more time near the sika deer speakers. Indeed, at least one female exhibited behaviors towards the sika deer speaker that were highly characteristic of female behavior during estrus when approaching a potential mate: focused attention and very close approach [53]. These two females (aged 3 and 4 years old) both had prior reproductive experience and were representative of the median age of females used in this study (median age = 4 years old). Overall, our results indicate that female red deer are not entirely adverse to novel male sika deer moans and thus, despite their divergence, male mating calls do not represent a solid pre-zygotic reproductive barrier between these species. However, female reactions to heterospecific male sika deer moans may alter considerably with increased familiarity and experience (i.e. as these species become more sympatric).

Our results suggest that the wide diversity of Cervinae male mating calls may not be a consequence of strong species discrimination mechanisms, but rather a consequence of divergent sexual selection pressures (e.g. selection for acoustic cues to size or dominance, selection for signals with improved environmental propagation, etc.) combined with geographic isolation and phylogenetic contingencies. Indeed, several experimental studies have confirmed the function of key acoustic properties of male red deer roars in female choice decisions [39], [43], [44]. In turn, female preferences for specific acoustic parameters in male sexual signals may result in failures of species discrimination when sexual signals of unfamiliar heterospecifics contain ‘attractive’ [9], [54], ‘exaggerated’ [43], [54], [55], or ‘novel’ male traits [56]–[58]. Male sika deer moans are much higher pitched than male red deer roars, and female red deer in estrus prefer male red deer roars with higher F0 (though the bases for this preference remain undetermined, [44]). Male sika deer moans may therefore be perceived as attractive, exaggerated, or novel versions of male red deer roars by some female red deer receivers, although with increased sympatry between these species, female red deer may improve their discrimination decisions for male mating calls through reproductive character displacement of female choice if fitness costs are associated with hybrid matings [18], [59]. Studies on captive animals show no obvious physiological disadvantage to hybrids [29], [60] and studies of free-ranging animals suggest that initial pairings between red and sika deer occasionally produce fertile offspring [34] and that female hybrids do not have lower pregnancy rates than the parent species [61]. However, to our knowledge the overall fitness of hybrids has not yet been systematically contrasted with that of either parent species. Finally, given the recent phylogenetic divergence of red and sika deer [24], [25], [34], [62], we cannot exclude the possibility that pre-existing sensory biases [63]–[64] may contribute to the observed variability in female red deer preferences for male calls of red deer vs. sika deer, either through attraction for ancestral, but subsequently lost, male traits (as in house finches, [65]) or through sensory exploitation of a pre-evolved female bias that developed before the male traits which may trigger it (as seen in swordtails, [56], [66], and túngara frogs, [63], [67]).

While most female red deer in our experiment preferred conspecific calls, some individuals displayed preferences towards heterospecific calls, possibly as a result of the mechanisms discussed above. Regardless of why some female red deer approach male sika deer moans, in the absence of a harem-holding male red deer, the act of moving towards a reproductively compatible heterospecific caller in the wild could lead to hybridization if the female does not oppose or successfully avoid mating attempts by a male sika deer. Inter-individual differences in mate choice or species discrimination have been correlated with a variety of factors such as the environment [19], [68], social context [69], [70], age [71], [72], condition [73], [74], experience [15], [75], personality [76], endocrinology [47], and genetic compatibility [77]. While many studies of behavioral responses focus on average population behavioral characterizations, assuming homogenous behavior within groups, this study highlights the importance of taking inter-individual variability into consideration to gain a better understanding of evolutionary processes [78]–[82].

### Conclusion

This study is an important step in investigating the role that vocalizations may play in intra- and interspecific mating decisions in the context of hybridization. We demonstrated that in a simulated situation where female red deer in estrus encounter both conspecific male red deer roars and novel heterospecific male sika deer moans, most, but crucially, not all females approached the conspecific call. The minority of females that spend more total time near the sika deer speaker may represent cases of incorrect species discrimination where hybridization could occur in the wild. Additionally, we highlight the potential importance of inter-individual differences in mate preferences. Additional studies are being conducted to quantify interspecific and intersexual differences in male and female behavioral and physiological responses to conspecific, heterospecific, and hybrid male mating calls.
